# A Case of Acute Metritis

**Published:** 1853

**Authors:** J. Hale

**Affiliations:** Fordsville, Ky.


ARTICLE III.—*A case of Acute Metritis.*—By DR. J. HALE, of Fordsville.

On the 9th inst. I was summoned to attend Mrs. P. (aged 21 years, sanguine-nervous temperament), whom I had delivered on the 2d, of her first child. She had a natural labor, and did exceedingly well up to the 7th, when on aiming to rise from bed, she felt (as she expressed it), something fall into the right side, which caused considerable pain and weight on lying on that side, which continued to increase up to the morning of the 8th, when she was taken with a chill that lasted for about three hours, followed by fever and an increase of the pain and weight in her side. She said the fever had continued up to the 9th, and the lochia had stopped entirely, and that she felt as if she were swelled considerably—felt great weight or heaviness on rising up or turning in bed.

On the 9th I made a digital examination and found the cervix uteri apparently healthy, though considerably flexed in consequence of the retroversion of the fundus. On examination through the walls of the abdomen, I found the fundus retroverted and lying to the right of the median line. I immediately introduced, with some difficulty, the index finger of my right hand into the os uterus, and grasped the fundus through the walls of the abdomen with my left hand, and reduced the retroversion with but little difficulty, after which she expressed herself as feeling some better. Her pulse was eighty-five per minute and full, her tongue dry and slightly coated, her bowels torpid, skin dry, and face flushed. She complained of great pain in her head and back, also great soreness in the uterine region on pressure. The peritoneum and lateral ligaments were entirely healthy. I bled her twenty ounces and ordered Hyd. sub Mit., grs. 10, Ipecac, grs. 3, Sulph. Morphine, half grain, to be followed in eight hours by Oleum Racini, half oz., Oleum Terebin, oz. 1, with fomentations to the uterine region.

On the 10th I saw her again, she expressed herself as doing well. The medicine had operated well, the fever had left her, and the lochial discharge had made its appearance again. She complained of no uneasiness whatever. On examination I found the uterus in situ, and the swelling nearly entirely absent. I ordered Ether Nitrici half oz., Vin. Antimony every three hours, to keep up diaphoresis, and discharged her, as I thought, doing well. On the 12th, 1 was called to see her again. The messenger informed me that she had, the evening previous, taken thirty drops of Sulphuric Acid—mistook it for the Vin. Antimony. In the course of an hour afterwards, he said, she complained of feeling very sick, and in a short time she took a diarrhœa, which lasted her all night, attended by great pain and tonnina, and that she took a chill in the morning, followed by fever and pain in the head and back, in short he said she was in the same condition as when 1 first saw her.

On my arrival 1 found her lying on her back, apparently in great distress. On examination 1 found her flooding freely, which had been the case for about half an hour.

Her extremities were cold, her face pale and contracted, her pulse about ninety, small and compressible. 1 ordered Acetate Plumbi, grs. 24, Gum Opiis, grs. 2, to be made into six pills; one to be taken every half hour. With her hips elevated on a pillow and hot bricks to her feet, 1 left her to return next day. On the 13th 1 found her lying in the same position 1 had left her. She informed me that the pills had stopped the flooding, though she felt some uneasiness from pain in her head and back, and she thought her womb was swelled again. She said she felt great soreness on firm pressure, and some pain from turning in bed.

On examination 1 found the uterus in situ, though very much congested, and very sensitive to pressure. Her pulse was eighty-five, small and corded, cheeks flushed, eyes prominent, &c.

1 bled her 12 ounces and ordered Hyd. Sub Mit., grs. 10, 1pecac, grs. 2, to be followed in eight hours by Oleum Racini, half oz., et Oleum Terebin 1 oz., and left her. On the 14th 1 saw her again, the medicine had operated well, the fever had abated, and she expressed herself as feeling quite well. On examination 1 found the uterus somewhat indurated and sensitive to firm pressure, but no symptoms of active inflammation. 1 ordered Ether Nitrici, half oz., Vin. Antimony, gtt. 15, Dover's Powder, grs. 5, every four hours, and a liniment to be rubbed over the uterus twice a-day, composed of Gum Camphor, 1 oz., Spts. Turpentine 1 oz., to be kept up as long as she felt the soreness and enlargement. 1 discharged her. She is now well.

The reason 1 have offered this case to the public is simply this, that it presented some phenomena that 1 did not fairly understand. First, whether or not the metritis was caused from the retroversion or rather retroflexion. 1n the first place, 1 am inclined to believe it was, from the fact, that she had an easy labor and did well up to the 7th. There was no placental adhesion. The placenta came away in a short time after delivery, with but little flooding. 1s it or is it not posssible that the retroversion of the fundus and flexion of the cervix would mechanically prevent the lochia from escaping? 1 am inclined to believe it would, from the fact that the cervix in this case was considerably flexed, and the os uterus apparently closed, in consequence of it. Now whether or not the retention of the lochia would produce inflammation, 1 am not able to say, though 1 believe it would, as it is evident that the uterus was in a phlogesed or congested condition, and it could not relieve itself through any other aperture save the os uterus, consequently inflammation set up. 1t is well verified that congestion is the produce of inflammation in all organs and tissues. In the second place, whether or not was the diarrhœa caused from the Sulphuric Acid, I believe it was, from the fact that her bowels heretofore, had been very torpid, and she could not assign any other cause than the acid, as she had used very light diet, and was slightly under the influence of Opii that was given her in the form of Dover's powder.

In the third place, whether or not was the flooding caused from the diarrhœa, I am induced to believe it was, as I could not assign any other cause for it. It is evident that the diarrhœa, by the irritation it produced in the colon and rectum, invited an influx of blood to the then irritated uterine organs, which is in all cases the primary cause of uterine hemorrhage. If that was not the cause, I cannot give any, as I never have seen or read of uterine hemorrhage making its appearance at that period after delivery—the thirteenth day.

FORDSVILLE, Ky., Oct. 24, 1853.

